# Radiation Tolerance Testing Methodology of Robotic Manipulator Prior to Nuclear Waste Handling

**DOI:** 10.3389/frobt.2020.00006

**Published:** 2020-02-06

**Authors:** Kaiqiang Zhang, Chris Hutson, James Knighton, Guido Herrmann, Tom Scott

**Affiliations:** ^1^Department of Mechanical Engineering, University of Bristol, University Walk, Bristol, United Kingdom; ^2^H.H. Wills Physics Laboratory, University of Bristol, Bristol, United Kingdom; ^3^Department of Electrical and Electronic Engineering, University of Manchester, Manchester, United Kingdom

**Keywords:** robot, manipulator, radiation, tolerance, gamma, waste, radioactive, exposure

## Abstract

Dramatic cost savings, safety improvements and accelerated nuclear decommissioning are all possible through the application of robotic solutions. Remotely-controlled systems with modern sensing capabilities, actuators and cutting tools have the potential for use in extremely hazardous environments, but operation in facilities used for handling radioactive material presents complex challenges for electronic components. We present a methodology and results obtained from testing in a radiation cell in which we demonstrate the operation of a robotic arm controlled using modern electronics exposed at 10 Gy/h to simulate radioactive conditions in the most hazardous nuclear waste handling facilities.

## 1. Introduction

The nuclear industry in the UK and worldwide increasingly seeks cost-effective methods to implement remote technologies to enable decommissioning of legacy waste treatment and handling facilities to reduce residual hazard, a practice highly recommended by the International Atomic Energy Agency (Iqbal et al., 2012). Whilst modern robots are in ubiquitous use in other industries, such as manufacturing, significant uptake is yet to take place in the nuclear industry which would benefit greatly from increased use of robotics, if implemented to carry out work too hazardous or difficult for human workers. Remote operations are required in legacy nuclear facilities for the purposes of inspection, characterization, cutting, dismantling, sorting, and segregating hazardous waste prior to the demolition of buildings. Commonly-encountered hazards in legacy nuclear facilities include aggressive chemical species, radioactive materials emitting alpha, beta or gamma radiation, and asbestos. Often an understanding of the nature and distribution of these types of hazards is required before decommissioning can take place, and so providing this improved situational awareness, and the ability to carry out tasks remotely is where new robotic technology can offer benefits to the industry.

A conservative regulatory framework and high safety standards make nuclear operators conservative in their approach to adopting new technology, and so any new systems must have their capabilities demonstrated and rigorously tested in a simulated environment prior to use. Many facilities at the end of their useful life were built in the 1950s and 60s, based on simple but effective technology.

The use of simple remote systems is not new to the industry, but uptake has been slow and restricted: Houssay (2000) describes several systems already trialed, including for routine monitoring and surveillance at Savannah River in the USA, and for cleaning of steam generators in generating plant at Indian Point 2 as far back as 1989.

Modern electronics enables more intelligent use of robotic systems, but this comes with its own challenges. It has been perceived by many in the industry that radiation will render any modern electronic components immediately inoperable, and so electronics have generally been avoided. Famously several of the robots used to explore the Fukushima Daiichi stricken reactor cores were subject to extremely high doses resulting in their failure very quickly. Such extremes of radiation are not common in the field of nuclear decommissioning, and so this paper aims to challenge the received wisdom that radiation and electronics do not mix. In the current work we demonstrate that it is indeed possible to use electronic technology in radioactive environments with successful outcomes. The notion that “no electronic equipment can survive radiation” and so cannot be used for any decommissioning task is not correct, and our experiments using a high activity cobalt-60 radiation source have shown that useful work can be done using sophisticated robots.

The radiation tolerance of many electronic components is of course of the utmost importance for robotic functionality (Garg et al., 2006). Often, researchers or manufacturers have attempted to make their electronic devices more radiation tolerant by altering the designs of integrated circuits, increasing the signal amplitudes or by simple shielding (Ferlet-Cavrois, 2011). In some instances however, the most cost effective solution for the use of electronics in radioactive environments is to use standard components, and plan for their replacement. Clearly, there are instances in which replacement would not be possible (e.g., space exploration or long term insertion in a reprocessing tank), yet by understanding the exposure environment posed by each application and the required component longevity, it is possible to develop appropriate solutions. Central to the assessment of each application is knowledge of the radiation tolerance of each component and also the system as a whole.

Many irradiation tests of electronic components examine individual integrated circuits or chips (for example, the work of Katz and Some, 2003; Nagatani et al., 2011; Ducros et al., 2017), whereas our trial sought to test the whole robotic arm, to better simulate what might happen in a real industrial environment containing highly radioactive material. A methodology is suggested for designing experiments to assess the system performance of an industrial robot whilst the robot is carrying out a dynamic task. In this way, it is possible to observe the degradation of the robotic system during its operation, and so the test provides a more meaningful assessment of operational challenges, compared to a performance assessment carried out on individual components or when the robot is stationary.

This is the very first work that assesses the system performance of an off-the-shelf industrial robot arm. It was not clear to the manufacturers whether the KUKA iiwa LBR800 would be operable at all when exposed to the levels of radiation contained within facilities handling Intermediate Level Waste (ILW). In this paper we describe an experiment to test the radiation tolerance of a robotic arm as an exploratory test to determine the off-the-shelf radiation tolerance of such a system, and to understand what improvements might be carried out to increase its suitability for decommissioning applications.

## 2. Methodology for Performance Assessment of Industrial Robots

Robotic systems containing electronic components are likely to suffer some form of damage causing altered functionality when exposed to radiation, and the effect will be related to the exposure dose. For many decommissioning tasks, the materials present emitting radiation will not be well-defined, and so measurement of the environmental dose rate may well be one of the robotic inspection tasks.

The effect of radiation on components is material dependent, and has been well-studied. Metal-oxide semiconductors (MOS) have electronic properties altered (Ma and Dressendorfer, 1989), elastomer materials used in seals can become embrittled (Wündrich, 1984), and optical components have been known to change their transparency and refractive indices over time (Brichard et al., 2001). Changes in the magnitude of measured errors in motor control were observed by Howard et al. (2018). Given enough radiation, these changes in mechanical, optical, or electronic properties can ultimately cause failure in susceptible components, potentially causing the robot to break down.

The impact of radiation on a robotic system can depend on its operational state, and a “stationary” assessment method may not demonstrate performance changes as an entire system. For systems operated in dynamic motion it is critical to guarantee the system performance of the whole robot (Aitken et al., 2018; Tsitsimpelis et al., 2019) to enable the robot to complete its tasks for a specific mission. Recently, different off-the-shelf industrial robots have been proposed, for example the iiwa 14 LBR820 suggested by Aitken et al. (2018) and the prototype systems funded by the UK Nuclear Decommissioning Authority (2019). The deployment of off-the-shelf robots avoids the need to design and manufacture special robotic arms for particular requirements, speeding up deployment on nuclear sites but most off-the-shelf industrial robots have not been tested in a radiation environment. For compliance with strict regulatory controls and to build safety case arguments, it is essential to qualify the system performance of an industrial robot arm during exposure from radioactive materials to reduce the risk of an accident.

This paper proposes a systematic methodology to assess the performance of an industrial robot consisting of the following steps:

1. Identification of the critical positions in the robotic arm containing (often electronic) components potentially susceptible to radiation exposure.
2. Planning the robot motion for specific applications considering any safety and physical constraints.
3. Measurement of exposure dose rates at each critical position.
4. Data collection and performance monitoring of available parameters and indicators during a repetitive motion of a planned trajectory whilst exposed to radiation.
5. Following an observed performance degradation, assessment of the point of failure using the “stationary” method.

Step 1. Due to high complexity of off-the-shelf industrial robots, it is often very hard to establish analytical models of the systems which may be influenced by radiation (Howard et al., 2018). However, it is possible to identify the components which are least radiation tolerant and estimate their failure dose through individual component irradiation testing. Typically, the control processors and the sensor's integrated circuits (ICs) are considered as the least radiation-hardened components, according to the results of stationary assessments (Katz and Some, 2003; Nagatani et al., 2011; Ducros et al., 2017). It might also be necessary to consider the degradation of other materials such as elastomer polymers (for structural integrity; Wündrich, 1984) and optical fibers (for device-to-device communication; Brichard et al., 2001).

Step 2. An industrial robot needs to be assessed in a radiation environment at a required dose rate over a given exposure time (Katz and Some, 2003; Nagatani et al., 2011; Ducros et al., 2017; Tsitsimpelis et al., 2019). This is easy to achieve in typical stationary assessments as individual electronic components can be exposed to a constant dose rate. For a closer simulation of real operation we recommend the use of a dynamic test, in which the robot undertakes a repetitive motion, providing firm evidence about the failure mode to be encountered during real operation. A comparison of operating parameters collected during each cycle of repeating actions during periods of exposure and prior to exposure can be used to show or predict changes in robot performance. The trajectory of the robot must avoid obstacles in the event of a malfunction, particularly with radioactive sources.

Step 3. In practice, exposure dose rates vary depending on the proximity of each component of the robot to the radiation source, and the differences can be dramatic. It is important to accurately measure the dose rate at locations of interest, particularly the less radiation-tolerant components, such that performance can be correlated with exposure dose. The dose rate at each position should be measured over multiple dynamic task-cycles, requiring a real time radiometric instrument such as a diamond radiation detector as described by Hutson (2018).

Step 4. All possible measurements related to the performance of a robotic system should be recorded for later analysis of degradation from radiation exposure. Howard et al. (2018) recorded all the input and output signals of each sensing and controlling component at a high sampling rate of 1.25 kHz, during their test exposure using X-rays. However, such hardware level signals are often difficult to access in industrial robots, but it may be possible to observe at least the control errors from robotic controllers.

A reference data set collected whilst moving in the planned trajectory, but prior to any exposure is desirable to enable analysis of any immediate effects radiation may have on a robot's operation.

Step 5. The last step for assessing the industrial robot is to run a stationary performance of the robot to identify the total radiation tolerance of the system at a configured dose rate until a system failure occurs (as in Nagatani et al., 2011; Ducros et al., 2017). As a result, the assessment step identifies the extreme total dose-tolerance of the industrial robot and any specific hardware/software issues limiting the overall system.

The proposed methodology has been used in the current study for assessing the system performance of a KUKA iiwa 7 LBR800 robot arm positioned ~1.60 m from a 20 TBq source of cobalt-60 (^60^*Co*).

## 3. Experimental Set-up

### 3.1. Robot Under Test

#### 3.1.1. KUKA iiwa 7 LBR800 Robot

**Figure 1:**
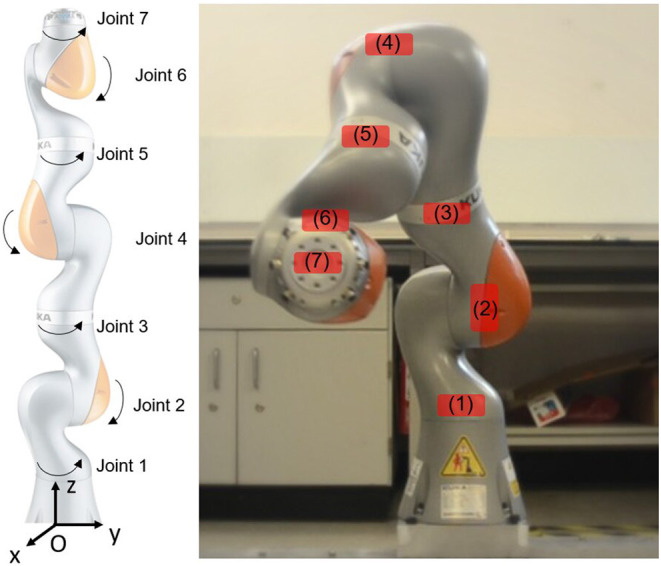
The KUKA iiwa 7 LBR800 robot used in radiation tolerance testing. The left image shows the robot in its “zero” position status, with all the robot joints at their initial zero positions. The positive directions of each joint are indicated. The right-hand photo shows the robot during a mock-up test in which a periodic trajectory was programmed. The red colored areas show mounting locations used for the radiation detector.

The KUKA iiwa 7 LBR800 robotic arm manufactured by KUKA Deutschland GmbH (2019) has been proposed for several uses in the nuclear industry, including decontamination of glove boxes. It has 7 rotational joints providing 7 degrees of freedom, and has a maximum payload of 7 kg with a 926 mm reach. The robot is highly flexible, allowing it to easily avoid obstacles. Figure 1 shows the location of the robot's joints. Within each joint, the robot has three different types of sensor to enable measurements of temperature, angular position, and force-torque. At each joint, two encoders used for angular position measurements, to achieve good positioning performance of its end-effector at milli-meter level. The use of two encoders provides some redundancy which could be used to detect sensor failure, and comparison of their feedback is used for a robot control safety mechanism. Force-torque sensors are used to detect any external forces applied to the robot, making it “human-safe” for work in which humans collaborate with the robot.

#### 3.1.2. Identification of the Least Radiation Tolerate Components

In each joint there are several electronic components such as the integrated circuits of motor drives, encoders, and torque sensors, which are all enclosed by an aluminum casing. These joint electronics constitute the locations of interest, and so dose rate measurements were made as shown in Figure 1. The most important of these was the “end-effector” responsible for carrying tools, grippers, and sensing packages. The end-effector is also likely to receive a higher dose than other components during operations dealing with radioactive material.

#### 3.1.3. Irradiation Test Source

**Figure 2:**
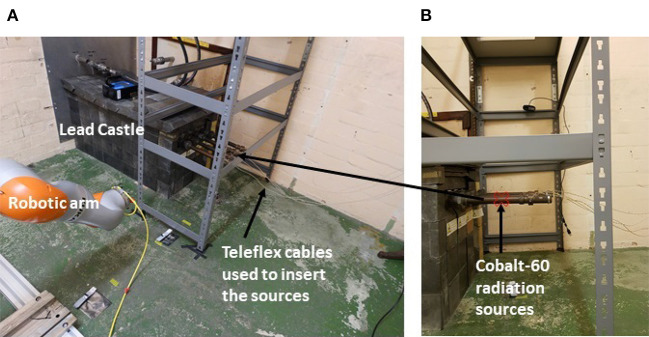
**(A)** KUKA iiwa robotic arm in front of cobalt-60 source tubes **(B)**.

**Figure 3:**
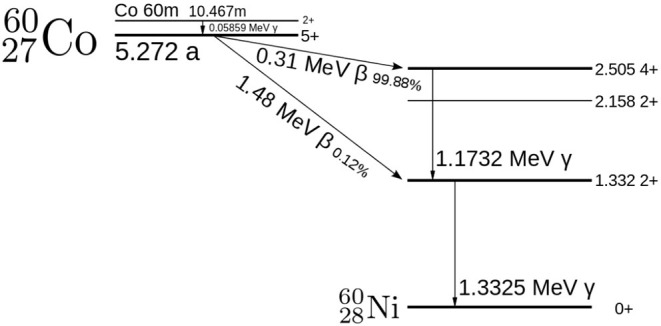
Beta decay of C2760o is followed by emission of gamma at 1.17 and 1.33 MeV from N2860i (Camp and Van Hise, 1976).

The radiation tests were carried out at the Medical Research Council (MRC) Harwell facility. As illustrated in Figure 2, the radiation source was made up of four ^60^*Co* sources with a combined activity of around 20 TBq. The ^60^*Co* sources produce intense gamma radiation via a decay to ^60^*Ni*, as shown in the decay scheme in Figure 3. The source can roughly be considered a point source located at the position marked in Figure 2B.

During decay, beta particles produced were attenuated by the source tube casings, leaving just gamma photons with energies 1.17 and 1.33 MeV. ^60^*Co* is used as a convenient radiation source because it is present in nuclear waste and emits photons close in energy to those emitted during the decay of ^137^*Cs*, the main contributor to the gamma emitted by long-lived nuclear waste. To expose the cell, the four ^60^*Co* sources were pushed with teleflex cables through containment tubes, passing into the cell via a lead castle into four protruding exposure tubes. The sources could be withdrawn at any time during the experiment.

### 3.2. Robot Trajectory Planning for Dynamic Performance Test

The robotic arm was anchored in place attached to a heavy base designed to stabilize the robot in the event of an unexpectedly high momentum should the robot control fail catastrophically. The robot was positioned so that all objects were out of reach as shown in Figure 2.

The robot's x-z plane was aligned with the sources and the source-tubes were parallel to the robot's y-axis (see the robot's original coordinates in Figure 1). The robot's original position was about 1.6 m from the center of the sources along its x-axis.

The robot is controlled to follow a defined trajectory, simulating the robot executing a repetitive task. The continuous motion of the robot arm is designed in a way to ensure each of the motors is active at all times. Therefore, this evaluates whether each joint could remain physically capable of moving during exposure (see results in section 4). The robot's end-effector is considered the most important component of the system, and has a target exposure of ~10 Gy/h. Ideally, the robot's end-effector would be controlled to follow an arc-trajectory retaining a constant distance toward to the assumed point source. Practically, this is not possible since very small changes in the distance from end-effector to source result in noticeable changes in dose rate. These changes were easily overcome using dosimetry operated at a frequency of 20 Hz.

From the center of the point source, the flux of gamma radiation decays as distance from the source increases following the inverse-square law. The robot's end-effector trajectory was planned to follow an arc-trajectory of radius 1 m from the assumed point source. The end-effector was moved along the arc-trajectory at a speed of 20 mm/s in a time period of 1 min, sweeping around the radiation sources in a repeating pattern. The trajectories were computed via a Jacobian matrix-based inverse kinematic algorithm as described by Meredith and Maddock (2004).

**Figure 4:**
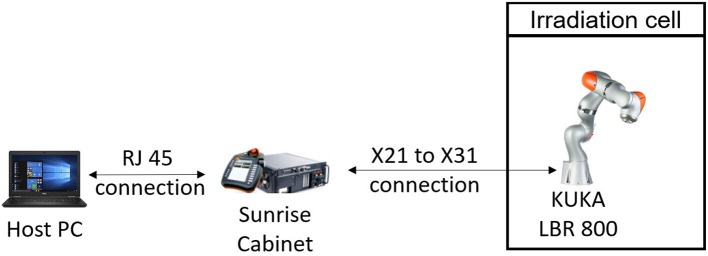
Set-up enabling the control and data recording of the LBR800 robot.

The robotic arm was connected by a robot control cable (X21-to-X31 cable) to a KUKA Sunrise cabinet control box, as shown in Figure 4. The control box was situated outside of the irradiation room (behind concrete walls), which meant it experienced little radiation dose. A host PC was connected via an RJ45 connection to the control box. In command of the 1-min cycle, the host PC instructed new position values for each joint to the control box, which passed these to the relevant joints. Specifically, a C++ control program was developed to enable the real-time communication between the standard host PC and the Sunrise Cabinet. The control program employed an application programming interface, called KUKA Fast Robot Interface (KUKA Deutschland GmbH, 2019). This design minimized the communication latency and, therefore, allowed for 100 Hz control/data-acquisition rate.

### 3.3. Dose Rate Measurement at Critical Positions

The primary purpose of the test was to understand radiation tolerance, and this required measurement of the robot's exposure dose. A real time measurement was required to accurately quantify the dose accrued prior to failure by each component of interest. A passive dosimetry measurement would not have been suitable for this test since the time to failure could not be predicted, and the failure could occur when experimenters were not present to withdraw the radiation sources and end the exposure.

The measurement required is particularly onerous for radiation detectors, since the exposure was sufficient to affect electronic performance. The most suitable available detector was a diamond radiation detector calibrated for air kerma dose rate measurement, a system already in use at Sellafield and described by Hutson (2018). This detector was chosen over other semiconductor detectors and scintillation detectors for its excellent radiation tolerance, and was already suitably calibrated for the target exposure dose rate. The detector used a single crystal diamond grown by chemical vapor deposition measuring 4.5 × 4.5 × 0.5 mm. The diamond detector system applies dose rates calibrations obtained using both caesium-137 and cobalt-60, and produces values for air kerma dose rates every 50 ms. Operating in current mode, the detector has no known upper dose rate limit and has proven extremely tolerant to gamma radiation.

**Figure 5:**
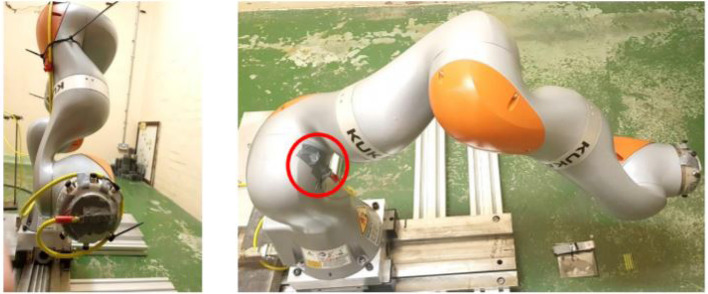
Exposure dose rates varied across the length of the robotic arm, hence the diamond detector was mounted at different locations on the KUKA iiwa robotic arm during the radiation tolerance test, to enable the dose rate measurements shown in Figure 7.

The detector was placed at a series of positions along the robotic arm in turn, to quantify the exposure of each bundle of electronic circuitry and sensors, as shown in Figure 5. For each position, real time dose rates were measured over 10 min. Once the exposure to each joint had been measured, the diamond detector was attached to the robot's end effector for the remainder of the experiment to continue real time radiometric measurement. The end effector component was subject to the largest radiation dose and it was of most interest for the test, because in a real nuclear decommissioning operation would hold any cutting tool or sensing package.

### 3.4. Identifying Robot Performance Degradation

**Figure 6:**
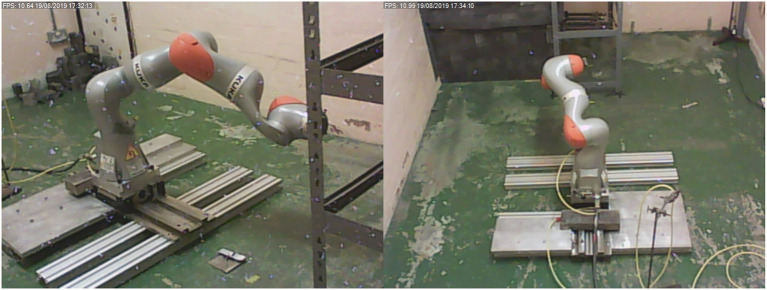
The robotic arm was observed during dynamic irradiation test using two webcams located inside the irradiation cell. Speckle may be observed in both images.

There were several indicators to help identify performance degradation and failure of the robotic arm:

1. Demanded and measured joint torque and position values were recorded in real time into a data spreadsheet at a sampling frequency of 100 Hz. Prior to the irradiation, the values were logged for the robot's arc trajectory for a reference set of values that could be compared to the values recorded during the irradiation. Any differences in the pre-irradiation and irradiation values would illustrate degradation in robot performance.
2. Two webcams recorded continuous video footage throughout the irradiation experiment. Example images are given in Figure 6, showing the footage could be used to monitor the robot in real time and identify any unexpected movements from the robotic arm.
3. The KUKA system displayed error messages of the robotic arm. It was expected that the robot's software would act to shut down the system for safety reasons in the event of a component failure.

In summary, we followed the methodology described herein consisting of: (1) identifying vulnerable components; (2) programming a repeating trajectory; (3) measuring the exposure dose rates of vulnerable components; (4) measuring degradation; and (5) observing system failure.

## 4. Results and Discussions

### 4.1. Exposure Dose Rate Measurement

**Figure 7:**
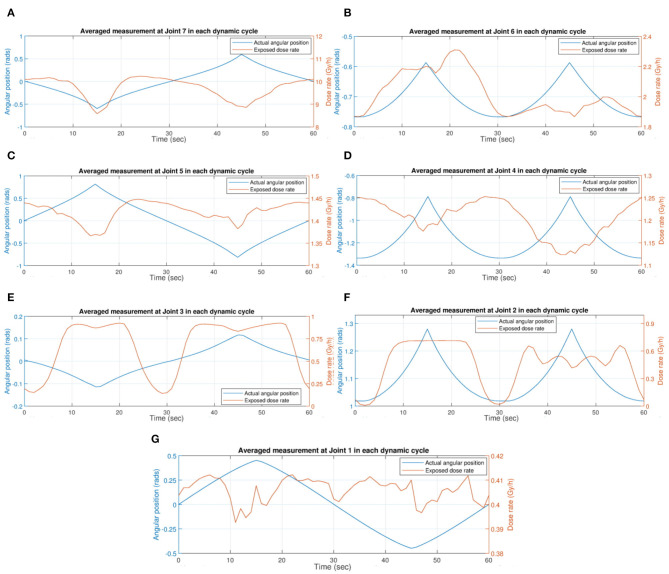
The averaged dose rate and angular position measurements of each dynamic assessment cycle (for 60 s) over 10 cycles. The angular position and exposed dose rates are shown for each joint: **(A)** at Joint 7; **(B)** at Joint 6; **(C)** at Joint 5; **(D)** at Joint 4; **(E)** at Joint 3; **(F)** at Joint 2; and **(G)** at Joint 1.

Necessarily at different distances from the radiation sources, and moving around an arc, each set of sensors and actuators was exposed to different fluxes of radiation. Therefore, for each location of interest on the robot a separate dose rate profile was measured, to characterize the exposure conditions for each joint. Measured using a diamond radiation detector, these dose rate profiles are shown in Figure 7.

### 4.2. Robot Performance up to Failure

During the initial dynamic experiment, the robot was controlled to follow the planned trajectory repetitively for ~6.3 h. This was a deliberately repetitive action, aimed to provide a detailed understanding of any changes in parameters occurring as a result of accumulated radiation damage. These chronic symptoms were important to understand in case they had any bearing on the overall control of the robot.

**Figure 8:**
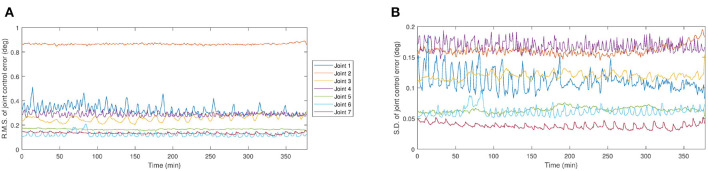
Evaluation of the angular position control performance of the robot at each joint. **(A)** The root mean square (R.M.S.) of the control error at each joints. **(B)** The standard deviation (S.D.) of the control error at each joints.

- No changes to the robot trajectory as a result of exposure were large enough to notice visually using the webcam images.
- No changes to the robot trajectory as a result of exposure were noticeable by changes in the dose rate profile by comparing a dosimetry profile at the beginning and end of the dynamic assessment.
- Subtle changes to the standard deviation of the Joint 2 control error began to occur after 5 h of this test (after a total of 8 h exposure) (shown in red in Figure 8).
- No other joints suffered the same increase in control error during irradiation, leading us to believe it may have been caused by a slight original defect in Joint 2, not necessarily caused solely by radiation damage.

### 4.3. Acute Failure

The robot failed during its stationary exposure, with the cause being a damaged optical encoder in the end effector (Joint 7). This component converts the angular position of the joint to a digital signal for feedback to the controller. The damaged component was diagnosed by the KUKA control system itself, with a message “encoder error torque sensor” and “safe axis position invalid,” and as a result the controller prevented further operation of the robot. Attempts to reboot, remaster, and the passage of time have been unsuccessful in recovering robot movement: this failed component was permanently destroyed.

### 4.4. Exposure Dose Rate and Total Dose

The dose rates measured by the diamond detector dosimetry system are shown for each joint in Table 1 for both dynamic and stationary assessments. These measurements have been used to calculate the overall exposure to each joint as detailed in Table 2.

**Table 1:** The air kerma exposure dose-rate at each robot joint, measured using the diamond dose rate system.

	**Exposed dose per dynamic cycle (Gy)**	**Dose rate during dynamic assessment (Gy/h)**	**Dose rate during stationary assessment (Gy/h)**
Joint 7	0.1619	9.71	9.68
Joint 6	0.0336	2.02	1.93
Joint 5	0.0237	1.42	1.41
Joint 4	0.0201	1.21	1.16
Joint 3	0.0113	0.68	0.92
Joint 2	0.0077	0.46	0.53
Joint 1	0.0068	0.41	0.41

**Table 2:** The air kerma exposure dose at each robot joint, measured using the diamond dose rate system.

	**During dynamic assessment (Gy)**	**During stationary assessment (Gy)**	**Overall exposed dose before failure (Gy)**
Joint 7	91.83	72.72	164.55
Joint 6	19.08	14.47	33.55
Joint 5	13.42	10.58	24.00
Joint 4	11.38	8.70	20.08
Joint 3	6.40	6.87	13.27
Joint 2	4.35	3.95	8.30
Joint 1	3.84	3.08	6.91

The uncertainty of the dose measurement is ±0.6 Gy in the dynamic assessment for about 9.3 h. Within the following static assessment, the exposure dose measurement has an uncertainty of ±0.49 Gy over a period of about 7.5 h. The system exposure dose has approximate ±1.09 Gy uncertainty.

## 5. Discussion

The KUKA LBR800 robot stopped operating after a large radiation dose of 164.55(±1.09) Gy to its end effector, and the component causing the failure was an optical encoder. The failure of this component was noted by the control software and smartPAD controller, which subsequently prevented the robot operating. The inbuilt smart software features were able to take excellent control of the situation, and we were able to demonstrate that once the encoder failed, Kuka's software locked down the robot in a safe state. This fail-safe mechanism in software would not have been possible in other more traditional types of robots using fewer electronic components, so this software approach should be considered as a significant safety benefit to any nuclear operator should such faults occur on a nuclear licensed site. Our test demonstrates that the standard safety features help ensure that a nuclear material handling accident due to a slowly failing system would not be possible.

It would be useful in a nuclear facility to maintain a cumulative dose reading on a joints (using diamond detectors or other similarly-sized miniature detectors) to ensure the system can be given preventative maintenance or component replacement, at say 75% of its dose-to-failure lifetime, rather than waiting for device failure at an inconvenient stage in a process.

The target dose rate of 10 Gy/h was chosen as a conservatively high value of exposure for ILW facilities, and would in reality more closely represent dose rates found in facilities dealing with high level waste. Contact dose rates encountered in ILW-handling facilities are generally 1 Gy/h and below, and so to simulate ILW facilities more closely it would be appropriate to use lower exposure doses for future testing programmes.

This paper presents a methodology to test off-the-shelf robots in radiation environments at a system level. Such system level tests are of significant benefits providing reference data to deploy the robot into practical operations, thereby building confidence that the system has potential to be used in radioactive environments. The system-level tests also allow for identifying the least radiation-tolerant component. Clearly, the system-level test is the necessary initial assessment in order to apply an off-the-shelf system in a radiation environment.

Note that investment required to buy complete off-the-shelf systems such as the robot tested in this work is significant. Therefore, although it is important to carry out system-level tests to build confidence in environments found in real applications, the cost of destroying large numbers of robots would be prohibitive. Hence, the following methodology is recommended to generate reasonable confidence of expected lifetime:

1. System level test to verify the off-the-shelf robot has the potential to satisfy initial design requirements in a radioactive environment.
2. Identification of particular components susceptible to radiation, through component-by-component analysis of irradiated robot.
3. Exposure of a statistically significant number of the identified susceptible components, allowing an estimation of system service life as a function of a variety of conditions, such as exposure at several dose rates, different temperatures and other robot load/operation modes. In a comparable study, two samples of each individual components were tested to develop a radiation hardened robot for the nuclear industry (Sharp and Decreton, 1996). In a good compromise between statistical rigor and cost, Oomichi et al. (2007) tested 7 to 30 samples of different components. For component-level testing, we recommend at least 20 samples be irradiated and analyzed to enable rigorous statistical analysis.

### 5.1. Recommendations for Further Work

Due to the conservative approach to utilizing new technology in the nuclear industry, it is of prime importance that the radiation tolerance of novel technology is understood fully prior to use. In this experiment the gamma radiation failure dose for the KUKA robot arm was 164 Gy. However, due to the probabilistic nature of photon interaction with matter, there is a probability (which is based on photon energy and electronic material) that when a photon is incident on an electronic device within the robotic arm, it will not be absorbed. Furthermore, the manufacturing process of electronic components produces a distribution of characteristics, so if the experiment were repeated, the failure dose may be higher or lower than we measured. Therefore, further irradiation tests both of several complete robotic arm systems would be useful to confirm the failure dose result. Clearly, this would have significant cost implications, so we recommend that instead, irradiation testing of a large number of the least radiation tolerant electronic components (e.g. the optical encoder) could be performed. This would still provide an accurate figure on the radiation tolerance of the whole system.

A second recommendation is to fix/replace the broken encoder in the end effector of the robot and attempt to restore the robot's full functionality. After the robot is remastered, more irradiation tests can be performed. This would be useful to industry as it would demonstrate that the robot can be fixed and redeployed, and it will provide more irradiation data for the arm.

Consideration of shielding of particularly vulnerable components inside the robot would increase the system's lifetime in a radioactive environment. It would be beneficial to trial some micro-shields around components such as the optical encoder in each joint, constrained by the impact of the additional weight from shielding which will reduce the possible payload of any sensors or actuators (e.g., grippers or cutters). Selective shielding could, at limited cost, substantially increase the lifetime of the robot.

Component replacement with radiation tolerant alternatives should be considered if the off-the-shelf robot was unsuitable. For example, the function performed by the optical encoders could be carried out by rotary encoders, which are known to be less susceptible to radiation damage. A cost benefit analysis would consider the added cost to implement the new components and the money saved by having an increased lifetime of the robot.

Small alterations in the software safety features within the robotic arm could be altered to allow the robot to recover should a joint fail whilst in the middle of undertaking a task. A robot with as many as seven joints has significant kinematic redundancy in the positions within reach, so it is possible for the robot to still complete its task without a joint in operation. This would extend the lifetime of the robot further, alternatively it could enable the robot to complete its immediate tasks and afterwards return to a safe state ready for corrective maintenance.

Future studies could model/simulate the damage caused to the robot's electronics using Monte Carlo modeling software such as the Geant4 package developed by CERN. Previous research similar to this has been performed for the application of radiation damage of electronics used in space by Feng et al. (2007) and Xiao et al. (2018). Such modeling would require more detailed knowledge of the distribution of radiation sources than is generally available for decommissioning nuclear facilities.

## 6. Conclusions

The current work has investigated the controlled exposure of a KUKA iiwa LBR robot to gamma radiation to determine its tolerance and performance in highly radioactive environments analogous to nuclear waste processing and storage facilities.

The robot was exposed to gamma radiation from a 20 TBq cobalt-60 source and displayed significant radiation tolerance, with failure occurring in an optical encoder after a cumulative exposure of 164.55 Gy over a period of 16.8 h.

The results indicate that force-torque robots, which offer an enhanced level of finesse for manipulating objects, are potentially viable for nuclear waste processing applications. Used in appropriate applications, robotic technology using modern sensing and control software has the potential to make a large impact in the sector in terms of cost savings, safety and shorter decommissioning timescales. Future work should consider alternate radiation-tolerant replacements for optical encoders, and also examine methods of micro-shielding of vulnerable components to enhance the performance lifetime of the robotic systems.

## Data Availability Statement

All datasets generated for this study are included in the article/supplementary material.

## Author Contributions

TS, CH, and KZ contributed conception and design of the study. CH organized the irradiation exposure and measured the dose rates. JK and KZ programmed the control, data collection of the robotic arm, performed the data processing, and statistical analysis. CH, JK, and KZ each wrote major sections of the manuscript. Both KZ and CH supervised JK. TS and GH are the principle investigators associated with this project. All authors contributed to manuscript revision, read, and approved the submitted version.

### Conflict of Interest

The authors declare that the research was conducted in the absence of any commercial or financial relationships that could be construed as a potential conflict of interest.
